# What Do Artificial Orthography Learning Tasks Actually Measure? Correlations Within and Across Tasks

**DOI:** 10.5334/joc.144

**Published:** 2021-01-13

**Authors:** Xenia Schmalz, Gerd Schulte-Körne, Elisabetta de Simone, Kristina Moll

**Affiliations:** 1 Department of Child and Adolescent Psychiatry, Psychosomatics and Psychotherapy, University Hospital, LMU Munich, Germany

**Keywords:** Learning, reading, test-retest correlation, Paired Associate Learning, Artificial Orthography Learning

## Abstract

Artificial Orthography Learning (AOL) may act as a possible candidate to model the learning of print-to-speech correspondences. In order to serve as an adequate task, however, we need to establish whether AOL can be reliably measured. In the current study, we report the correlations between the learning of two different artificial orthographies by the same 55 participants. We also explore the correlation between AOL skill and other participant-level variables, namely Paired Associate Learning (PAL) performance, word and nonword reading ability, and age. We find high correlations between learning of two different artificial orthographies. Correlations with reading fluency and PAL are low. These results leave questions about the link between reading acquisition and AOL. At the same time, they show that AOL ability can be reliably measured and justify its use for future studies.

Reading research often aims to assess which cognitive processes differ across individuals varying in reading ability, or how learning to read is affected by reading instruction and various characteristics of an orthography. The long-term aim of such research is to identify person-level or environmental conditions which pose challenges to children learning to read, and ultimately to counteract these conditions with targeted interventions. Studying reading acquisition in a natural environment can be challenging. First, it is time consuming, as reading is acquired across many years. Second, children differ in their pre-existing knowledge about orthography by the time they start learning to read. This makes it difficult to exclude co-varying factors as alternative explanations to any observed patterns. To circumvent these issues, numerous researchers have turned to Artificial Orthography Learning (AOL) tasks (Aravena, Snellings, Tijms, & van der Molen, 2013; Aravena, Tijms, Snellings, & van der Molen, 2017; Bishop, 1964; Bitan & Karni, 2003; Byrne, 1984; Law et al., 2018; Maurer, Blau, Yoncheva, & McCandliss, 2010; Taylor, Davis, & Rastle, 2017; Taylor, Plunkett, & Nation, 2011; Yoncheva, Blau, Maurer, & McCandliss, 2010). These studies aim to recreate the conditions underlying reading acquisition by teaching (mostly adult) participants a new orthography within a limited number of sessions. The orthographies consist of pseudowords (or real words from the participants’ language; Taylor et al., 2017; Yoncheva et al., 2010), which are written with unfamiliar symbols. The symbols generally correspond to phonemes (or other phonological units) which exist in the participants’ languages.

AOL experiments are proposed to act as a model of learning the relationship between print and speech (though some studies have also included semantics; Taylor et al., 2011; Taylor et al., 2017). According to theories of reading acquisition, the learning of print-to-speech correspondences is a *sine qua non*: once children learn how to derive a phonological output from an unfamiliar written word, they can use this decoding process to read almost any word that they have not seen in its written form before (Castles, Rastle, & Nation, 2018; Share, 1995). The importance of this decoding process is further demonstrated by the finding that children with dyslexia show disproportional difficulties with pseudoword reading, which is an indicator of decoding skills (Rack, Snowling, & Olson, 1992; but see also Van den Broeck & Geudens, 2012).

Having an experimental paradigm to simulate the acquisition of print-to-speech correspondences in a controlled setting is therefore very useful. It would allow us to study participant-level and item-level characteristics which affect the ease of learning, as well as instructional factors that may either facilitate or impede the learning task. The main advantage of AOL is that the experimenter has full control over various properties of the orthography (item-level characteristics), the participants’ pre-existing knowledge of this orthography (which should be close to zero), and the way in which this orthography is taught. Control over item-level characteristics is important, because in psycholinguistic research, variables tend to be correlated: thus, if we want to identify the influence of a particular language-level variable on the ease of reading acquisition, it is difficult to manipulate this specific variable while keeping constant all other linguistic properties that we know to, or may found out in the future to influence reading processes (Rayner, Pollatsek, Drieghe, Slattery, & Reichle, 2007; Yap & Balota, 2009). Similarly, it is also informative to study effects of participant-level characteristics on reading acquisition: in a natural setting, one does not know how participant-level characteristics may have affected the prior knowledge or cognitive skills that are specific to print-to-speech correspondence learning.

Studies of children learning to read in a real language are irreplaceable, if we want to understand how reading acquisition happens. However, small-scale experiments, such as the AOL paradigm, may be useful to focus on specific aspects of reading acquisition. A fruitful approach to isolating item- and participant-level variables which affect reading acquisition, or optimal teaching strategies, is to start with a controlled experiment, which can be used to formulate a specific hypothesis, and subsequently verify this hypothesis with a large-scale developmental study, using methods that are targeted at addressing this hypothesis.

This approach would rest, though, on the assumptions that (1) performance on an AOL task reflects a print-to-speech-correspondence-learning-skill which can be reliably measured, and (2) the type of cognitive processes that underlie this learning are similar to the cognitive processes that enable reading in children learning to read in a language which they already speak. In the current paper, we aim to test these assumptions. First, we assess the reliability of AOL tasks, by examining correlation between the same adults learning two different artificial orthographies. Second, we explore correlations with other participant-level variables, namely word and nonword reading fluency and the ability to learn unsystematic visual-verbal associations (Paired Associate Learning, hereafter: PAL).

## METHODS

### PARTICIPANTS

The participants were 60 staff or students at universities in south Germany. All were native German speakers. They participated in individual sessions lasting up to 2 hours in exchange for 20 Euro vouchers or course credit. From 60 participants, 5 did not complete all tasks due to fatigue. These five participants are excluded from all analyses. Of the remaining 55 participants, the average age was 24 years (SD = 8, min = 18, max = 56).

### READING ABILITY

Reading ability was tested with a standardised 1-minute reading fluency test (SLRT II; Moll & Landerl, 2010), which includes a word and a nonword reading list. Norms have been collected for German adult readers for both the word and the nonword list. The first list contains 156 words and the second list contains 156 pseudowords, and participants are given 60 seconds to read aloud as many items as they can. For the word reading test, the participants of the current study read, on average, 123 words per minute (SD = 13, min = 88, max = 151), which corresponds to an average percentile score of 56.4 (SD = 27.2, min = 9, max = 97). For the nonword reading test, participants read, on average, 79 nonwords per minute (SD = 16, min = 59, max = 112), corresponding to an average percentile of 53.7 (SD = 25.1, min = 4, max = 99).^1^

### ARTIFICIAL ORTHOGRAPHY LEARNING: FIRST TASK (SMALL ORTHOGRAPHY)

The first AOL task consisted of 12 CVCV-structured words, which were made of four consonants (phonemes /b/, /g/, /j/, and /n/) and three vowels (phonemes /o:/, /u:/, and /y:/). Each phoneme was represented consistently by a symbol. The symbols were chosen from the lower-case symbols of the BACS font, a set of artificial characters which are similar to letters in terms of their visual complexity (Vidal, Content & Chetail, 2017). The items and their phonetic transcriptions are listed in Appendix A. For each item, we created an audio file, where the pseudoword was recorded by a German native speaker.

The training procedure is closely based on Taylor et al. (2011). Even though the participants are trained on whole words, they extract knowledge about the underlying symbol-sound correspondences, as shown by their ability to subsequently read aloud novel items written with the same symbols (i.e., the ability to *generalise* the symbol-sound knowledge). The training procedure occurred in two phases: In an exposure phase, the participants were presented with the written and spoken form of each item simultaneously (1 exposure of each of the 12 items). Their task was simply to repeat the spoken form. In the training phase, participants saw the written form of each of the 12 items, and were instructed to read it aloud. If they were unsure, they were encouraged to guess. At the end of a five-second interval, the participants heard the correct pronunciation through a set of loudspeakers. The experimenter scored the participants’ accuracy by verifying the correspondence between the participant’s pronunciation and the feedback at the end of the trial. If the participant obtained an accuracy rate of greater than 70% (i.e., 9/12 items or more), the training was considered complete. If this accuracy threshold was not reached, the entire set was presented to the participant again. If the participant did not achieve threshold accuracy after 5 runs through the training block, the test was terminated and the experimenter moved on to the next task.

### PAIRED ASSOCIATE LEARNING (PAL) TASK

The PAL task was designed to have a similar training procedure as the AOL tasks. Participants were presented with ten different shapes (e.g., rhombus, empty circle, spiky circle), that had been randomly matched to disyllabic (CVCV) pseudowords (e.g., /’be:t͡sa:/). The pseudowords were recorded by a German native speaker. The full list of shapes and the corresponding phonology is listed in Appendix A.

Initially, participants went through an exposure phase: They saw each shape and simultaneously heard its “name”. The instructions were simply to repeat what they heard. In the training phase, each shape was presented, one at a time, and the participants were instructed to say its “name”. At the end of the trial, after a five-second interval, they heard the correct pronunciation and the experimenter scored the accuracy of the participant’s response. The training block was repeated until the participant obtained an accuracy rate of at least 70% (i.e., 7/10 items). If the participant did not reach this threshold after 5 runs through the training block, the task was terminated and the experimenter moved on to the next task.

### ARTIFICIAL ORTHOGRAPHY LEARNING: SECOND TASK (LARGE ORTHOGRAPHY)

The second orthography included 36 items which had a CVC structure. There were 12 consonant symbols and 6 vowel symbols. Eleven of the consonant symbols were consistently pronounced as the phonemes /f/, /ʃ/, /k/, /l/, /m/, /n/, /p/, /ʁ, /s/, /t/, and /t͡s/. One of the consonant symbols had a context-dependent pronunciation: it was pronounced as /h/ when it occurred in the first position of a pseudoword, and as /ç/ when it occurred in the final position, and another symbol was pronounced as /z/ when occurred in the first position and as /s/ when it occurred in the final position. This contextual changes was necessary for the artificial orthography to comply with the phonotactic rules of German: The phoneme /h/ never occurs in final word positions (when words end with the letter *h*, e.g., *nah, sah*, the letter *h* serves to indicate a long vowel), and /ç/ rarely occurs at the beginning of words. The vowel phonemes were the monophthongs /e:/, /o:/, /u:/, and /y:/, and the diphthongs /aɪ/ and /ɔʏ/; each was represented consistently by a single symbol. In the German orthography, diphthongs are generally represented by two letters (/aɪ/ most frequently as *ei*, /ɔʏ/ as *eu*). Similarly, the phoneme /ʃ/ is generally represented by the three-letter grapheme *sch*. Due to the mapping of these phonemes to a single symbol in the artificial orthography (as well as introducing the position-specific rule relating to the symbol corresponding to /h/, /ç/), participants had to extract regularities that did not exist in their native orthography. The symbols were, again, chosen from the BACS font (Vidal et al., 2017). We took symbols from the upper-case font, thus ensuring that there was no overlap with the previous artificial orthography. Each item was recorded by a native German speaker. The full list of items and their transcriptions can be found in Appendix A.

The exposure and training phase were identical to the first AOL task. Training was continued until participants gave the correct pronunciations to at least 26/36 items (>70% accuracy). If the participant did not reach the threshold after 5 runs through the training block, the task was terminated. The maximum number of runs through the training block was determined in a pilot study, where 20 staff or students at a university in south Germany who did not participate in the current study learned the orthography to criterion in 5 run-throughs or less.

Compared to the previous artificial orthography task, there was an additional test phase, the generalisation phase, which was conducted after training was concluded (provided that the participant reached the accuracy threshold). This test phase aimed to measure the degree to which participants had developed generalisable knowledge of the symbol-sound correspondences. Here, participants were presented with pseudowords which had not occurred during training, but consisted of the same symbols. The instructions were to read aloud each pseudoword, and to guess if they were unsure. The participants’ responses were scored offline by a German native speaker.

## RESULTS

**Figure 1:**
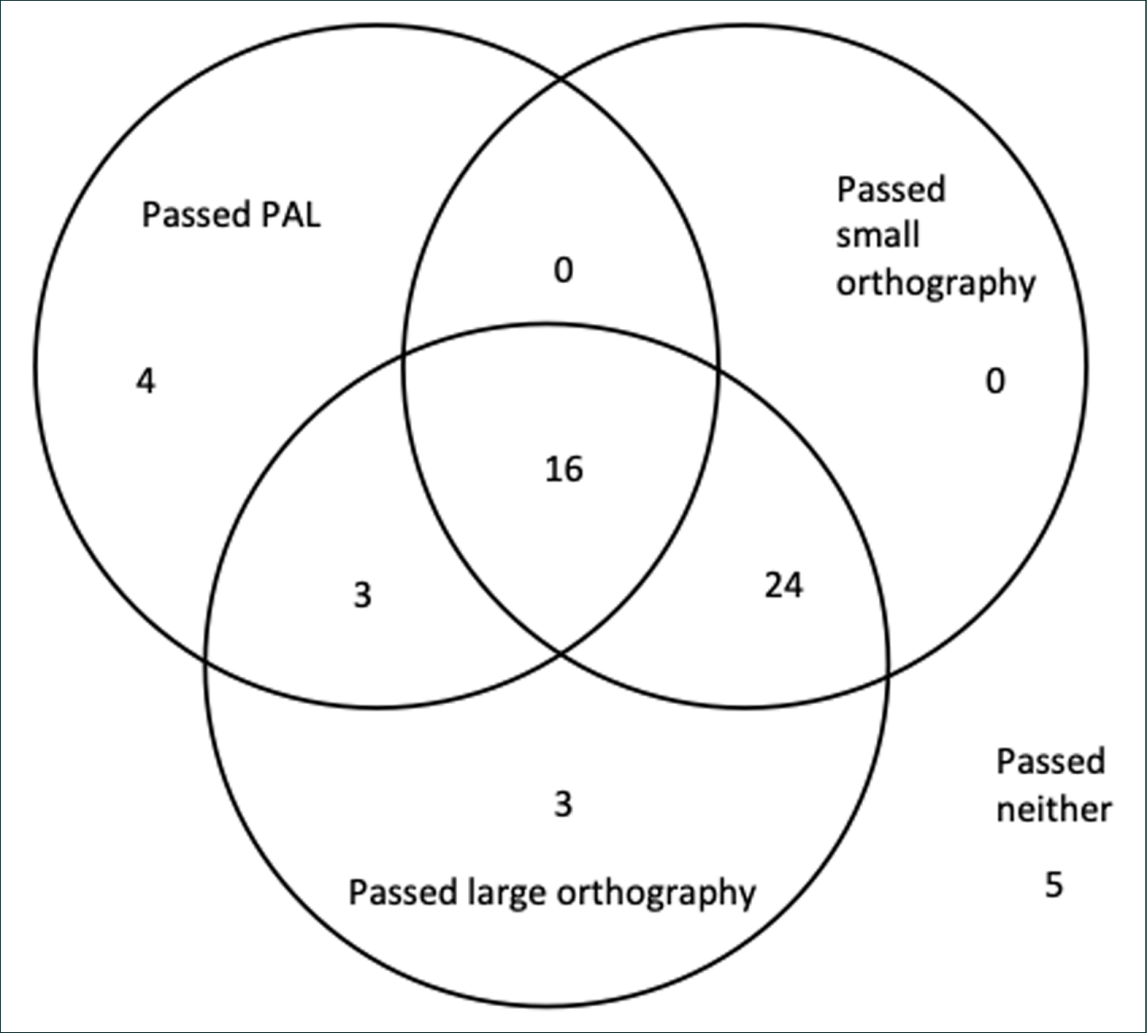
Summary of how many people passed the training phase for each constellation of tasks.

Not all participants learned the two artificial orthographies and/or the PAL task to criterion. Sixteen participants reached the criterion for all three tasks, 5 did not reach the criterion for any of the three tasks; others learned only one or two of the tasks to criterion. The constellation of these is summarised in a Venn Diagram in ***Figure 1***.

### CORRELATIONAL ANALYSES

In order to explore the correlations between the reading and learning tasks, the original plan was to correlate the number of run-throughs which participants needed to learn the materials to criterion against each other and against reading ability, as this was the dependent variable used by Taylor et al. (2011). For example, the first participant reached criterion after 3 runs through the training block for the first orthography, after 5 run-throughs for the PAL task, and after 5 runs through the second orthography. However, a large number of participants did not reach criterion on all three tasks (see ***Figure 1***), meaning that such an analysis would require us to exclude more than half of the data that we collected. However, there are other variables that can be calculated based on our data. We perform a multiverse analysis (Steegen, Tuerlinckx, Gelman, & Vanpaemel, 2016): In a multiverse analyse, the data are analysed in as many different ways as possible and all analyses are reported. This allows the analysist to look for converging evidence across approaches and to identify stable patterns in the data and scenarios under which the effects cannot be observed, while retaining maximum transparency about the analysis choices.

Along with the Pearson’s correlation coefficients (*r*), we report and interpret Bayes Factors (BF), which quantify the degree to which the observed correlation is compatible with the alternative hypothesis (H_1_) for the presence of a correlation, over the null hypothesis (H_0_) of no correlation. The BFs were calculated with the software JASP (Love et al., 2015), using the default parameters, where the prior for H_1_ is defined as a beta-distribution with a width parameter 1, which corresponds to an uninformative prior (i.e., there is no *a priori* expectation about some range of *r*-values being more likely than another range of the same length). The BF is a ratio: values of BF > 1 provide evidence for H_1_, and values BF < 1 provide evidence for H_0_ (i.e., for the absence of a correlation). By convention, we interpret BF > 3 or < 1/3 as some evidence for H_1_ or H_0_, respectively; BF > 10 or < 0.1 as strong evidence, and BF > 100 or < 0.01 as very strong evidence. BFs between 1/3 and 3 are considered to provide equivocal evidence, where more data is needed in order to draw conclusions.

In the first set of analyses, we report the correlation coefficients for the 16 participants who completed all tasks, using the number of run-throughs to quantify the performance on the training task. The correlations between all tasks are presented in ***Table 1***.^2^

**Table 1:** Correlation coefficients and Bayes Factors between the tasks, including only participants (*N* = 16) who learned all tasks to criterion. *Note*: SLRT = Salzburger Lese- und Rechtschreibtest (standardised reading test), PAL = Paired Associate Learning, Orth1 = first artificial orthography, Orth2 = second artificial orthography. Bold cells indicate BF > 3, and italics indicate BF < 1/3.


		SLRT WORDS	SLRT NONWORDS	PAL TRAINING	ORTH1 TRAINING	ORTH2 TRAINING	ORTH2 GENERALISATION

Age	r	–0.25	–0.37	0.33	*–0.04*	–0.14	0.21

	BF	0.47	0.78	0.63	*0.31*	0.35	0.41

SLRT Words	r	—	**0.59**	–0.34	0.14	–0.47	0.50

	BF	—	**4.36**	0.65	0.35	1.46	1.90

SLRT Nonwords	r		—	**–0.61**	–0.34	–0.41	0.25

	BF		—	**5.66**	0.65	0.99	0.46

PAL training	r			—	0.13	0.45	–0.40

	BF			—	0.35	1.30	0.92

Orth1 training	r				—	0.29	–0.03

	BF				—	0.53	0.311

Orth2 training	r					—	**–0.64**

	BF					—	**8.09**


With the subset sample, we find some evidence for the presence of three correlations (marked in ***Table 1*** in bold font): (1) Participants who are faster at reading words are also faster at reading nonwords, (2) participants who are faster at reading nonwords need fewer runs through the PAL training, and (3) participants who learn the second orthography faster show superior performance on the generalisation task for the same orthography. We find some evidence against one correlation (marked in ***Table 1*** in italics), namely between the learning of the first artificial orthography and age.

Taking into account all 23 participants who passed the PAL task, the pair-wise correlation between the number of PAL runs and nonword reading speed (number of correct pseudowords on the SLRT) was *r*(22) = –0.25, BF = 0.47 (equivocal evidence). Taking into account 45 participants^3^ who passed the second orthography training phase, the correlation between the number of blocks needed to reach criterion and generalisation accuracy was *r*(44) = –0.49, BF = 50.50 (strong evidence), suggesting that participants who learned the orthography more quickly also had higher accuracy at applying this knowledge to a set of novel words written in the same symbols.

**Figure 2:**
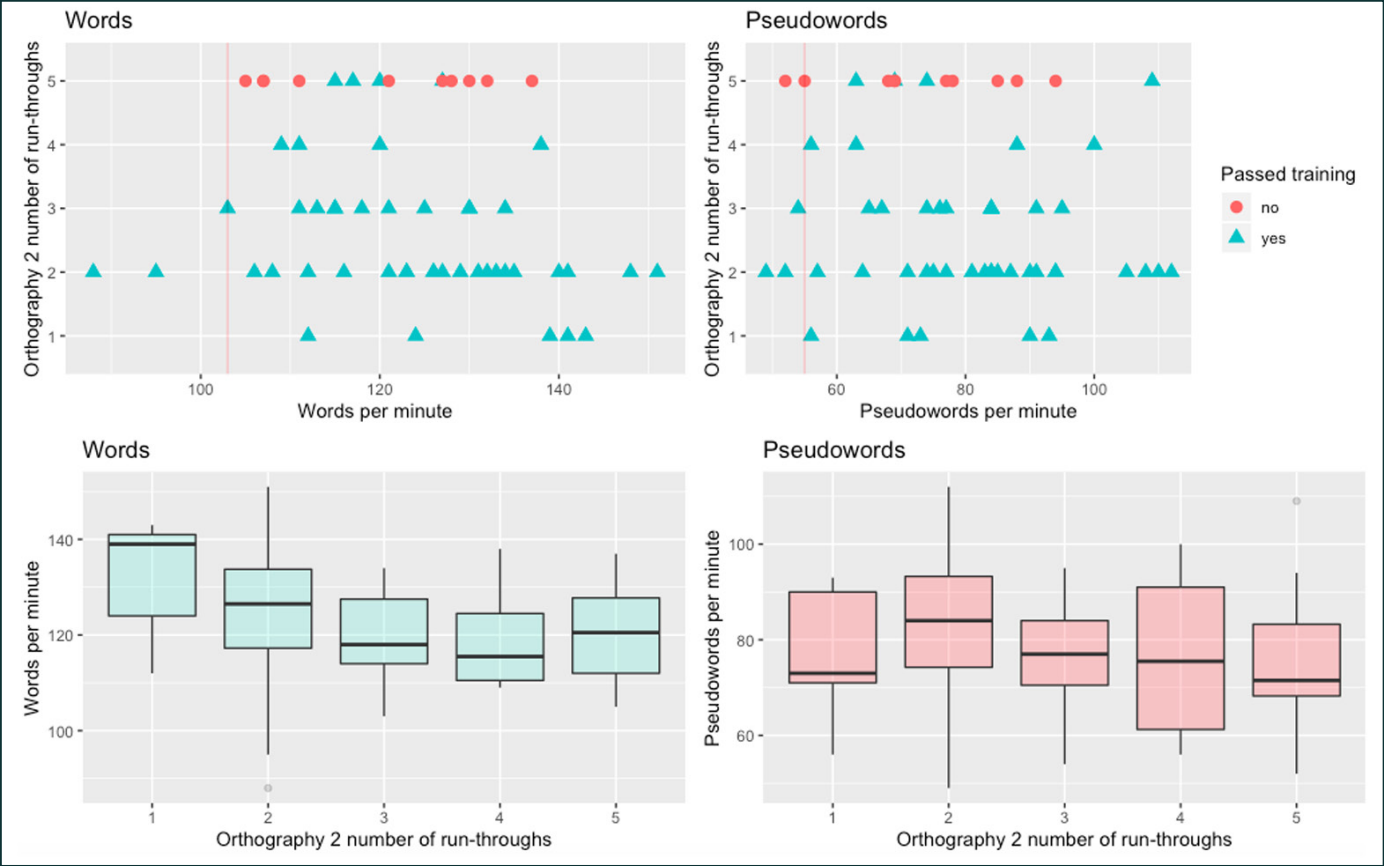
Scatterplot of reading ability (words and pseudowords) and the number of training run-throughs that the participants completed for the second orthography, coloured by whether or not they passed the training phase after the fifth run-through. The vertical line represents the 15^th^ percentile cut-off according to SLRT adult norms.

Following up on the relevant correlation between reading ability and the speed of AOL learning, we calculated the correlation, for 46 participants who passed the second orthographic training phase, between the number of run-throughs, and their SLRT word and pseudoword reading scores. The correlations were *r*(45) = –0.24 (BF = 0.66; equivocal evidence) for words, and *r*(45) = –0.07 (BF = 0.20; some evidence for the null hypothesis) for pseudowords. The lack of a strong relationship is further demonstrated in ***Figure 2***: the upper panels show a scatterplot between the number of run-throughs and reading ability, including the participants who did not pass the training task (for whom the number of run-throughs equals to 5). The lower panels show the average reading speed for participants who needed different numbers of run-throughs to achieve criterion accuracy.

Given that many participants were excluded for the above analyses, it is desirable to establish whether an alternative measure of training success can be used, which would allow us to include the data of all participants. One such measure could be the accuracy for each of run through the training blocks. All participants completed the first block, but many participants performed at floor level (14, 23, and 7 participants out of 55 had an accuracy of 0% for the first training block of the PAL, first orthography, and second orthography, respectively). At the later blocks, the number of participants dissipates as they reach the criterion and discontinue with the training. It can be assumed, however, that they would have performed equally well or better in the subsequent block had the training continued.^4^ Therefore, we copy-pasted the accuracy for the last block which a given participant completed to all following blocks for the same participant. The correlation matrix for the data excluding the imputed data can be found in the supplementary material (*https://osf.io/muhve/*, file “CorrelationMatrix_NoImp.xls” in the “Supplementary_analyses” folder), and relevant differences between the results with and without the imputed values are noted in the text below.

In line with a multiverse analysis approach, we examined the robustness of the above results by correlating performance in each training block of the second orthography against all other variables which are theoretically interesting (results reported in ***Table 2***). To assess whether there is a correlation between the training phases of Artificial Orthography 1 and Artificial Orthography 2, we correlated training accuracy across blocks. Here, presence of correlations between the two orthographies is mostly supported, with the exception of correlations with the training accuracy on the first block of the first artificial orthography. Correlations between Blocks 2–5 of Orthography 2 and Blocks 4–5 of Orthography 1 are strongest, with *r* coefficients between 0.68 and 0.86, and all BF > 90,000. These positive correlations suggest that participants who are fast at learning the first orthography are also fast at learning the second orthography. To verify the stability of these results, we also calculated the pairwise correlation coefficients between the accuracy on each block of Orthography 1 and Orthography 2, while excluding the imputed values (see supplementary analyses, *https://osf.io/muhve/*). The correlation coefficients do not change substantially, though discrepancies arise for the later blocks, due to the greatly reduced number of participants for whom we have non-imputed data.

**Table 2:** Correlations between accuracy on the second artificial orthography for each training block and all other variables, *N* = 55. *Note*: Pearson’s correlation coefficient, Bayes Factor in brackets. Bold font signifies evidence for the presence of a correlation, italics signifies evidence for the absence of a correlation. Orth2 = the second artificial orthography, Orth1 = the first artificial orthography, PAL = Paired Associate Learning, SLRT = Salzburger Lese- und Rechtschreibtest (standardised reading test).


ORTH 2 BLOCK	ORTH 1 BLOCK 1	ORTH 1 BLOCK 2	ORTH 1 BLOCK 3	ORTH 1 BLOCK 4	ORTH 1 BLOCK 5	PAL BLOCK 1	PAL BLOCK 2	PAL BLOCK 3	PAL BLOCK 4	PAL BLOCK 5	SLRT WORDS	SLRT NONWORDS

Block 1	0.18 (0.38)	**0.36** **(5.52)**	**0.43** **(36.42)**	**0.59** **(10,268)**	**0.54** **(796.20)**	*0.07* *(0.19)*	*0.12* *(0.24)*	*0.12* *(0.25)*	*0.06* *(0.18)*	0.21 (0.54)	0.26 (1.04)	*–0.10* *(0.17)*

Block 2	0.23 (0.70)	**0.45** **(46.94)**	**0.57** **(3,079)**	**0.81** **(>90,000)**	**0.75** **(>90,000)**	0.17 (0.34)	*0.11* *(0.23)*	*0.10* *(0.22)*	*–0.04* *(0.18)*	*0.14* *(0.28)*	0.19 (0.42)	*0.12* *(0.24)*

Block 3	**0.33** **(3.12)**	**0.48** **(153.22)**	**0.56** **(2,784)**	**0.82** **(>90,000)**	**0.86** **(>90,000)**	*0.07* *(0.19)*	*0.04* *(0.17)*	*0.00* *(0.17)*	*–0.08* *(0.20)*	*0.12* *(0.25)*	*0.10* *(0.22)*	*0.10* *(0.22)*

Block 4	0.29 (1.75)	**0.42** **(24.12)**	**0.52** **(521.39)**	**0.74** **(>90,000)**	**0.82** **(>90,000)**	*0.04* *(0.17)*	*0.05* *(0.18)*	*0.00* *(0.17)*	*–0.03* *(0.17)*	0.18 (0.40)	*0.08* *(0.19)*	*0.16* *(0.32)*

Block 5	0.29 (1.55)	**0.39** **(10.19)**	**0.48** **(140.02)**	**0.68** **(>90,000)**	**0.74** **(>90,000)**	*0.04* *(0.17)*	0.07 (0.56)	*0.01* *(0.17)*	*–0.02* *(0.17)*	0.19 (0.43)	*0.06* *(0.19)*	0.19 (0.42)


In contrast, correlations between training accuracy (accuracy for different training blocks, number of run-throughs needed to reach criterion) for Orthography 2 and PAL training accuracy are mostly not supported, with 21 out of 25 BFs providing evidence for the absence of a correlation. There is also mostly evidence against a correlation between Orthography 2 training accuracy and word reading speed (3 out of 5 BF < 1/3) and pseudoword reading speed (4 out of 5 BF < 1/3).

It is also theoretically relevant whether PAL training performance is correlated with reading ability, given that previous studies have studied PAL in relation to developmental dyslexia (Hulme, Goetz, Gooch, Adams, & Snowling, 2007; Litt, de Jong, van Bergen, & Nation, 2013; Litt & Nation, 2014; Litt, Wang, Sailah, Badcock, & Castles, 2018). This appears to be the case from the subset data in ***Table 1***, but not from the analyses including all participants who passed the PAL training task. Across the five training blocks of the PAL task, the correlation with word reading was 0.07 < *r*(54) < 0.19, all BF < 0.38, and with pseudoword reading, 0.19 < *r*(54) < 0.23, and 0.40 < BF < 0.68. Thus, the evidence for a relationship between PAL and reading speed was numerically positive (higher accuracy on the training blocks corresponded to increased reading fluency), but mostly equivocal.

Next, we looked at the relationship between the accuracy on the generalisation task of the second orthography and the other variables. Taking into account the 45 participants from whom we had generalisation data, accuracy on this task was correlated with Orthography 2 training accuracy on each of the blocks, 0.43 < *r*(44) < 0.48, all BF > 12. For a correlation between the generalisation task and training accuracy on the other artificial orthography task, there was evidence for the absence of a correlation for Blocks 1 to 3; –0.04 < *r*(44) < 0.13, all BF < 1/3, and equivocal evidence for Blocks 4 and 5; *r*(44) = 0.36, BF = 3.00, *r*(44) = 0.28, BF = 1.00. Between generalisation and PAL training performance, the evidence was against a correlation (Block 1 and 5) or equivocal (all BF < 2). There was no evidence for or against a relationship with reading ability, for words *r*(44) = 0.30, BF = 1.12, and for nonwords, *r*(44) = 0.16, BF = 0.33.

In a final set of analyses, we examined whether some items from the AOL task were more difficult to learn than others. The first orthography contained only one-to-one mappings, where furthermore each symbol had a corresponding Latin letter in the German orthography. The second orthography contained one context-sensitive rule that is not present in the German orthography or phonology: the same symbol mapped onto /h/ at the beginning of pseudowords and /ç/ at the end of pseudowords. Furthermore, diphthongs, which are represented, in the German orthography, by two letters (/ɔʏ/ and /aɪ/ as *eu* and *ei*, respectively), were represented by a single phoneme in the current orthography. Similarly, the phoneme /ʃ/ is represented by a trigram in German (*sch*), and by a single symbol in the artificial orthography.

To test whether participants made more mistakes for certain types of items, we transcribed their responses for the final block of the training task and for the generalisation task for the second AOL task. During testing, for each response, an audio recording was created for the interval of 3 seconds (training) and 5 seconds (generalisation). Unfortunately, this turned out to be insufficient for the training task: for 36% of the trials, the participant’s response was cut off, meaning that it was impossible to score as correct or incorrect. Among the transcribed responses, 74% were correct, which is in line with the threshold criterion of 70% for passing the training block. In the generalisation phase, 8% of the trials were invalid due to an early cut-off of the recording. A cut-off response suggests a higher reaction time, and thus, may be a useful measure, in addition to accuracy, to quantify the difficulty which the participants had with the particular item. We therefore conducted two sets of analyses: (1) on the number of correct responses over the number of responses which were valid, and (2) on the number of correct responses over the total number of trials. Between-item comparisons were conducted with a Bayesian *t*-test in R, using the package BayesFactor (Morey & Rouder, 2014), with the default prior settings, where the alternative hypothesis is a Cauchy distribution centered on zero and with a width parameter of 0.707.

For the percentage of correct responses over the number of valid responses, a Bayesian *t*-test showed some evidence for the absence of a difference between the training and the generalisation block (for training, average = 74.4%, SD = 8.9%; for generalisation, 75.9%, SD = 9.1%), BF = 0.30. For the training data, we found equivocal evidence for the presence of an effect associated with the context-sensitive rule (BF = 1.52), for an effect of the presence of a diphthong (BF = 0.58), or for an effect of the phoneme /ʃ/ (BF = 0.47). In the generalisation data, we found strong evidence for a group difference, depending on whether the item contained (average accuracy = 63.9%, SD = 10.5%) or did not contain (average accuracy = 77.5%, SD = 7.7%) the context-sensitive rule (/h/ in the beginning; /ç/ at the end of words), BF = 10.3. There was equivocal evidence, in the generalisation data, for an effect of the presence of diphthongs (BF = 0.72) or for an effect of the phoneme /ʃ/. We obtained converging results for the percentage of correct responses over the total number of trials, with some evidence for an effect of the context-sensitive rule in the generalisation data, BF = 3.6, and equivocal evidence for all other effects.

In addition to the sublexical characteristics, it is also possible that lexical characteristics affect the ease with which items are learned. Both for the training and the generalisation items, we computed the Phonological Levenshtein Distance (PLD) 20 (Yarkoni, Balota, & Yap, 2008): the average number of phoneme additions, substitutions or deletions needed to get to the 20 most similar words. The lower the PLD20, the more similar the pseudoword is to real German words. We correlated the percentage of accurate responses (both over the number of valid trials and over the total number of trials) with the PLD20 measure, for the training and generalisation items. We found mostly equivocal evidence against the presence of a correlation (|*r*| <0.27; BF < 0.7), and evidence against a correlation between PLD20 and the number of correct responses over the overall number of trials, BF = 0.22.

## DISCUSSION

In the current study, we took a multiverse analysis approach to exploring the relationship between performance on AOL and other tasks. Instead of frequentist significance testing, we interpreted the correlations with Bayes Factors, which indicate the extent to which the data is compatible with a hypothesis of a correlation over the hypothesis of no correlation. This allowed us to determine some stable results, both when it comes to the presence and the absence of correlations. The results are relevant to researchers seeking to use the AOL paradigm to study item-level, participant-level, or instructional factors affecting learning performance and generalisation.

We found a stable and high correlation between training performance of the Artificial Orthography 1 and Artificial Orthography 2 (*r* ≈ 0.75). This suggests that training performance is relatively stable across time, thus validating the use of this task as a reliable indicator of a participant characteristic. This suggests that, from a methodological perspective, the AOL task is well-suited to studying individual differences and behavioural correlates of this task. This conclusion is limited to the AOL paradigm as implemented in the current study: other studies have used different designs, for example, by teaching participants individual symbol-sound correspondences rather than encouraging them to extract these from the presentation of whole word forms (Aravena et al., 2013; Aravena et al., 2017; Law et al., 2018), or teaching participants to read words that exist in their native language, written in a novel script (Taylor et al., 2017; Yoncheva et al., 2010). It is unclear how such modifications would affect the relationship between AOL and reading ability, its psychometric properties, or if it measures the same learning processes as our version of the AOL task. However, it is noteworthy that there were differences between our Orthography 1 and Orthography 2. The two orthographies differed in the structure of the pseudowords (disyllabic CVCV pseudowords vs. CVC-monosyllables), the number of items and correspondences that were to be learned (with both more items and more symbol-sound correspondences in the second orthography), the consistency of the symbol-sound mapping, with position-specific rules changing the pronunciation of some symbols in the second orthography, and the presence of orthography-to-phonology mappings that contradicted the letter-sound knowledge from the participants’ native orthography (e.g., that one symbol instead of two letters mapped onto the diphthong /aɪ/, which is represented in the German orthography as the digraph *ei*).

Unexpectedly, not all participants were able to learn the orthography to criterion: 9 out of 55 participants were not able to learn the second orthography after 5 run-throughs, even though the orthography and the training procedure were closely based on a previous study (Taylor et al., 2011). The high rate of participants who did not learn the artificial orthography is comparable to a recent study, which adapted the paradigm of Taylor et al. (2011) to two AOL studies in Italian (Schmalz, Mulatti, Moll, & Schulte-Körne, 2019): here, across two experiments, 35% and 32% of participants failed to reach the threshold of 70% after 10 runs through the training block. Thus, across studies, participants show substantial variability in the speed at which they are able to learn the orthography. This variability is also evident within our study: five of our participants were able to reach the threshold during the first run through the training block, thus it is not the case that the orthography is too hard to learn for participants in general. We tentatively suggest that this high degree of variability may be the reason for the discrepancy in the learning completion rate in our study and that of Taylor et al. (2011): with the high variability in learning performance observed in the current study, we also expect substantial variability across samples in different studies.

AOL performance was not correlated with any of the variables that we collected, however, untested factors such as attention, motivation, experience learning other languages or orthographies, or language ability more generally may have influenced the current results. While this opens up a number of research questions about individual differences which may be responsible for the variability in AOL, caution is recommended for studies looking at item-level or instructional differences using a between-subject design: unless one controls for learning ability, or unless the sample size is large, the between-subject variability may result in group differences that are unrelated to the manipulation. This between-subject variability might pose challenges for studies which use a between-subject group design, but note that it does not affect our conclusion about the psychometric properties of the AOL task. As long as the variability between participants is constant for different AOL tasks (which we found to be the case in the current study), the conclusion is that some participants are consistently faster at learning artificial orthographies than other participants, even if, across the board, some orthographies are more difficult to learn than other orthographies. It is worth noting that the relatively high correlation between the two AOL tasks was found despite slight differences in the characteristics of the symbol-to-speech-sound correspondences.

We did not find a correlation between AOL and reading fluency. This may have implications for treating the AOL task as a model of reading acquisition: if both reading acquisition and AOL rely on the same cognitive processes, we would expect at least some evidence that faster readers are also better at learning the artificial orthography. However, there are alternative explanations for the lack of a relationship, mainly related to our use of adult participants. First, it is possible that the ability to extract and learn to apply symbol-sound correspondences affects reading ability mostly at the early stages of reading acquisition, as it should be specifically linked to the learning of print-to-speech correspondences. After children learn the print-to-speech correspondences of their orthography, other factors affect their reading progress. Learning the print-to-speech correspondences is an important first step in reading acquisition, but it is insufficient to develop fluent and automatised reading. Thus, by adulthood, reading speed may be only minimally affected by the participants’ decoding ability in early childhood. This is likely to be the case, especially for a transparent orthography such as German, where children learn the print-to-speech correspondences relatively quickly, and further individual differences associated with reading ability are manifested in reading speed rather than error rate (Landerl & Wimmer, 2008; Wimmer, 1993). Supporting the notion that reading ability in adulthood is driven by factors other than decoding ability, previous studies have found an increasing dissociation, across age, in reading comprehension and decoding tasks (e.g., Foorman et al., 2015; Garcia & Cain, 2014; Storch & Whitehurst, 2002).

Second, it is possible that adult participants approach the task of learning symbol-sound correspondences in a different way compared to children who learn to read for the first time. Specifically, adults might be engaging in a symbol-to-symbol memorisation task: rather than attempting to memorise the pronunciation of a given symbol, they might map the symbol onto their pre-existing orthographic representation of the sound (i.e., the letter(s) corresponding to the phoneme in the native orthography). While we cannot evaluate this possibility using the currently reported data, one anecdotal observation suggests that this might be the case, at least for some participants: our items included the diphthong /aɪ/, which, in German, is most frequently spelled by the two-letter grapheme *ei*, where the pronunciation of this two-letter grapheme does not correspond to the pronunciation of the individual letters (/e:/ and /i:/, similar to the English two-letter grapheme *sh*, which does not map onto the phonemes /s/ and /h/). Several participants, during the training procedure, mispronounced the symbol corresponding to /aɪ/ as /e:i:/, suggesting that they mapped the symbol onto a familiar orthographic (letter) representation, and subsequently converted this orthographic representation into sounds. As this observation is merely anecdotal, future research is needed to confirm it. In the current experiment, we did not find any evidence to suggest that participants are affected by a situation where a phoneme which maps onto several letters in German and only onto one symbol in the artificial orthography. However, in the generalisation phase, participants struggled with an inconsistent correspondence, even though its pronunciation was systematic when its position was taken into account. Whether this is attributable to the inconsistency of the symbol-to-speech-sound correspondence, or whether it is also affected by the inconsistency with the German orthographic representation of these phonemes, remains an open question.

As a third explanation, it is also possible that we would get a closer relationship if the format of the AOL task differed. The paradigm of Taylor et al. (2011), on which we based the current study, was conceptualised from a connectionist perspective of reading acquisition (Plaut et al., 1996). Here, knowledge of the print-to-speech-sound correspondences is extracted from teaching the system based on an input consisting of written word forms and an output consisting of the words’ pronunciations. In children, this process is likely to succeed a phase when they are explicitly taught letter-sound correspondences. The cognitive processes underlying this type of learning, and its relative importance compared to explicit letter-sound correspondence learning and other reading-related skills, are still unclear. It is possible that the ability to explicitly learn letter-sound correspondences has a greater effect on subsequent reading acquisition: in this case, an AOL task where participants are explicitly taught the pronunciations of each symbol (akin to Aravena et al., 2013; Law et al., 2018) may show stronger correlations with reading ability.

As a fourth explanation, our experiment may have had too few participants to detect a true but small effect. ***Table 2*** shows a medium-sized correlation between the number of run-throughs for the second orthography and word reading ability (*r* = –0.47) and the bottom left panel in ***Figure 2*** further suggests that a trend in the expected direction (i.e., participants with faster word reading ability needed fewer runs to achieve criterion accuracy).

In the current experiment, there was mostly evidence against a correlation between AOL and PAL. This suggests that the AOL task captures different cognitive processes than simply the rote memorisation of visual-verbal associations. How exactly these cognitive processes differ remains a question for future research. Alternatively, one could argue that the lack of a correlation may be explained, as above, if participants learn to associate a given symbol with the orthographic representation of its co-occurring phoneme. In this case, AOL would involve matching the visual symbol (e.g., ↓) against an orthographic representation corresponding to the phoneme (*sch*) rather than against the phoneme (/ʃ/). This would make AOL a visual-visual matching task, while PAL would be a visual-verbal matching task. However, this is unlikely to fully explain the lack of a correlation, because a previous study comparing learning performance across modalities found a correlation of about *r* = 0.3 between visual-verbal and visual-visual PAL (Litt et al., 2013).

It is tempting to draw a comparison between the AOL task and the learning of sublexical print-to-speech correspondences on the one hand, and between PAL and the learning of sight words on the other hand. In the domain of reading, it is well established that the learning of sublexical correspondeces and lexical knowledge depends, at least partially, on different cognitive skills, as nonword reading, measuring the ability to apply print-to-speech correspondences, and irregular word reading, measuring sight word knowledge, can be dissociated (Castles & Coltheart, 1993). Indeed, visual-verbal PAL has previously been linked to the ability to learn new written word forms (Wang, Wass, & Castles, 2016), and has been shown to predict irregular word reading ability but not nonword reading ability (Hulme et al., 2007). Thus, future developmental studies may want to assess whether there is a specific relation between AOL and nonword reading in children, as well as a link between PAL and sight word reading.

When it comes to the relationship between PAL and reading ability, the results were mostly equivocal. Thus, we cannot draw conclusions either about the absence or the presence of this correlation. The correlations between PAL and nonword reading were *r* ≈ 0.2 across different measurements, and between PAL and word reading *r* ≈ 0.15 across different measurements. These estimates may serve as guidelines for power calculations of future studies, if they want to provide evidence for or against a correlation between PAL and reading speed in an unselected sample of adult readers.

The current study opens questions for future research. As the first limitation of the current study, we have little information about the psychometric properties of the PAL task. While we showed that performance on the two AOL tasks correlate, suggesting good psychometric properties, it is possible that the PAL task (or our implementation thereof) would show worse psychometric properties, which could be an explanation for the weak or absent correlations with the reading task and with the AOL tasks. Second, the approach which we took in analysing the data was data-driven rather than hypothesis-driven, in the sense that we first analysed the data and then drew conclusions based on the most stable patterns of results. This exploratory approach provides a base for future, confirmatory approach.

In conclusion, the current study found that the AOL task is a reliable measure of the ability to extract symbol-sound correspondences from an artificial orthography. However, it is still unclear how this symbol-sound learning ability relates to reading ability and other cognitive processes. As we found no correlation between AOL and reading speed, future research is needed to establish whether there are shared cognitive processes between the AOL task and reading acquisition. Performance on AOL is distinct from the ability to memorise arbitrary visual-verbal mappings, which further opens questions about the cognitive learning processes which distinguish the learning of visual-verbal mappings which underlie orthographies from visual-verbal mappings in different contexts. Previous studies have used AOL tasks to assess what information is learned by the participants, and how item- and instructional-level variables affect learning performance (e.g., Byrne, 1984; Taylor et al., 2017). Establishing the reliability of this task further strengthens the results of these previous studies, and encourages the use of this paradigm in future studies.

## DATA ACCESSIBILITY STATEMENT

We would like to thank Erik Henzler and Arlette Kosecki for their help with data collection and transcribing the audio responses, and Veronika Jäger for recording the audio-stimuli. We are grateful to the reviewers for their helpful comments. The raw data, materials, and analysis scripts can be found here: *https://osf.io/muhve/*. This project was supported by a “Freies Wissen” stipend to XS from Wikimedia Deutschland, VolkswagenStiftung, and Stifterverband.

## ADDITIONAL FILE

The additional file for this article can be found as follows:

10.5334/joc.144.s1 Appendix A. Items for the first Artificial Orthography Learning task.

## Footnotes

The SLRT reading scores are comparable with previous results from a similar sample (Schmalz, Moll, Mulatti, & Schulte-Körne, 2019). We did not ask participants about childhood history of dyslexia or other disoders; however, several participants had very low reading scores (2 participants with <15^th^ percentile on the word reading task, 4 participants on the pseudoword reading task, and 1 on both). Therefore, the possibility that some participants may have a history of developmental dyslexia cannot be excluded. We did not specify dyslexia as an exclusion criterion, because the aim was to get a wide range of reading ability to maximise the chance to find meaningful correlations.

Note that, within the frequentist framework and with 16 participants, correlations with |*r*| > 0.5 reach significance at the 0.05-level.

Forty-six participants have completed the Artificial Orthography 2 task (see ***Figure 1***). However, due to an experimenter’s error, the audio recordings for one participant in the generalisation task were missing.

To test this assumption, we calculated how often a participant’s accuracy regressed rather than improved from one training block to the next. In the first artificial orthography, there were 8 instances of a participant’s performance regressing between one training block and the next, compared to 88 instances where performance continued to improve. In the second artificial orthography, there were no regressions. In the PAL task, accuracy regressed 24 times, as opposed to increasing 102 times.
