# Supplementary material for: What Do Artificial Orthography Learning Tasks Actually Measure? Correlations Within and Across Tasks
Source: J Cogn. 2021 Jan 13;4(1):7. doi: 10.5334/joc.144 (PMC7805385; doi:10.5334/joc.144)

Appendix A

Items for the first Artificial Orthography Learning task

/bo:’gu:/ 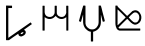

/gu:’jy:/ 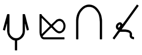

/jy:’nu:/ 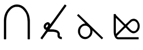

/nu:’go:/ 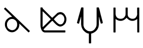

/go:’by:/ 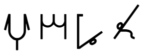

/by:’no:/ 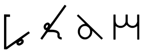

/no:’ju:/ 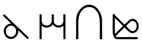

/ju:’ny:/ 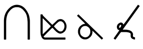

/ny:’bu:/ 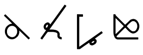

/bu:’gy:/ 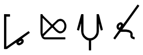

/gy:’jo:/ 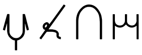

/jo:’bo:/ 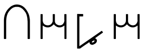

Items for PAL task

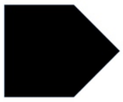	/’be:t͡sa/
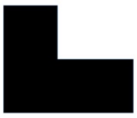	/’da:bo/
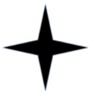	/’do:mu/

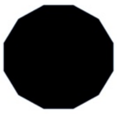	/’ga:bu/
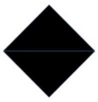	/’gi:fu/
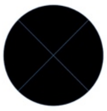	/’je:li/
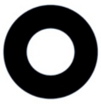	/’ku:bi/
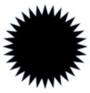	/’lo:vu/
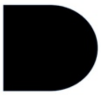	/lu:to/
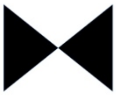	/’tu:po/

Items for the second Artificial Orthography Learning task

Training

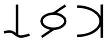 /kaɪf/

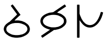 /zaɪm/

/taɪp/

/naɪl/

/faɪç/

/laɪʃ/

/pe:ʁ/

/me:s/

/t͡se:t/

/ʁe:n/

/he:k/

/ʃe:t͡s/

/nø:f/

/tø:m/

/mø:p/

/hø:l/

/zøç/

/lø:ʃ/

/ʃo:ʁ/

/po:s/

/fo:t/

/t͡so:n/

/fo:k/

/lo:ʁ /

/ʁy:f/

/hy:m/

/ky:p/

/ʁy:l/

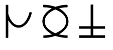 /my:ç/

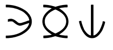 /t͡sy:ʃ/

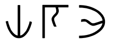 /ʃɔʏt͡s/

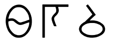 /ʁɔʏs/

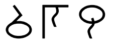 /zɔʏt/

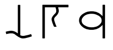 /kɔʏn/

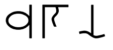 /nɔʏk/

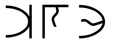 /fɔʏt͡s/

Generalisation

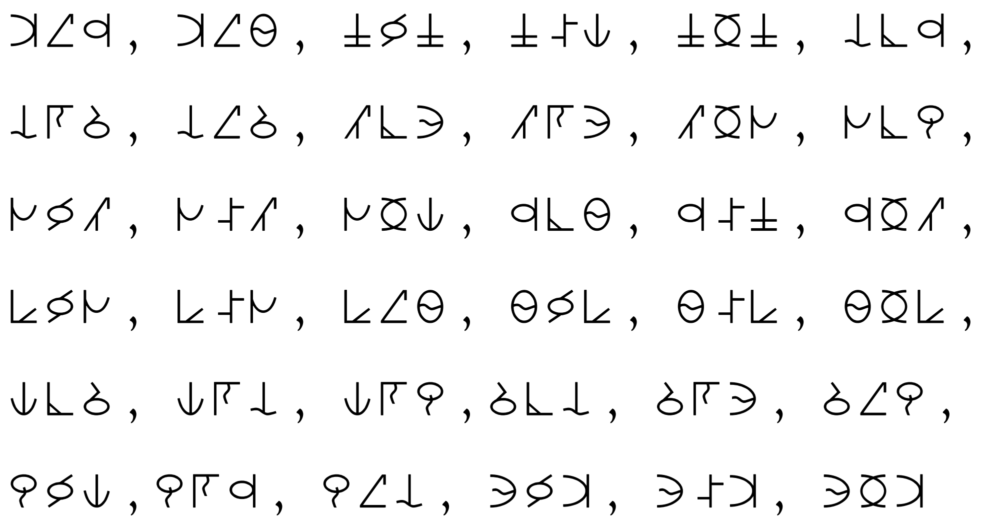

Supplement: Appendix A. — Items for the first Artificial Orthography Learning task. [file joc-4-1-144-s1.pdf]
